# Neutrophil extracellular traps, released from neutrophil, promote microglia inflammation and contribute to poor outcome in subarachnoid hemorrhage

**DOI:** 10.18632/aging.202993

**Published:** 2021-05-08

**Authors:** Zeng Hanhai, Qin Bin, Zhou Shengjun, Li Jingbo, Guo Yinghan, Cai Lingxin, Cao Shenglong, Zhou Hang, Chen Huaijun, Zhuang Jianfeng, Peng Yucong, Fu Xiongjie, Yu Xiaobo, Tan Xiaoxiao, Li Jianru, Gu Chi, Yan Feng, Chen Gao

**Affiliations:** 1 Department of Neurological Surgery, The Second Affiliated Hospital, Zhejiang University School of Medicine, Hangzhou, China; 2 Neurosurgerical Intensive Care Unit, The Second Affiliated Hospital of Zhejiang University School of Medicine, Hangzhou, China; 3 Department of Neurological Surgery, Ningbo First Hospital, Ningbo, China

**Keywords:** neutrophil, neutrophil extracellular traps, microglia, subarachnoid hemorrhage

## Abstract

Evidence indicates that neutrophil has promoted inflammation in several central nervous system diseases. However, whether the peripheral blood levels of neutrophils are associated with the functional outcome after subarachnoid hemorrhage and its potential mechanism remain unclear. In this study, we showed that neutrophil levels in peripheral blood were higher in patients with subarachnoid hemorrhage (*P* < 0.001) than in healthy subjects. Neutrophil levels were positively associated with Hunt and Hess grade (*P* < 0.001) and modified Rankin Scale scores at 3 months after SAH (*P* = 0.008). In terms of the mechanism, neutrophil extracellular traps markedly increased the proinflammatory subtype transition of microglia. After treatment with DNAse I, the proinflammatory subtype transition of microglia involving CD16 positive and IL-1β positive microglia was limited (*P* < 0.05). This mechanism was also verified *in vitro*. These results indicate that the existence of neutrophil extracellular traps, released from neutrophils after subarachnoid hemorrhage, can shift microglia toward a more proinflammatory phenotype and contribute to neuroinflammation and poor outcome in subarachnoid hemorrhage.

## INTRODUCTION

In central nervous system disease, peripheral immune cells, especially neutrophils, are closely related to the CNS [1–4]. Neutrophils are innate immune cells and play a key role in immune defense [5]. In recent years, research on neutrophils in pathogen clearance, immunoregulation and disease pathology has made great progress [5–8]. Neutrophil extracellular traps (NETs), reticular chromatin structures, have become a new area of intense research focus in biology [9–12]. In an inflammatory state, NETs can cause tissue damage [10, 11, 13–15]. In neurodegenerative diseases, stroke, and infection, NETs in human blood samples has been found to be positively or negatively associated with clinical outcome [16–18]. However, the role of neutrophils in SAH is still not fully explored.

Previous studies have shown that inflammation plays a pivotal role in subarachnoid hemorrhage (SAH), [19–23] and systemic inflammation attends SAH-induced injury [24, 25]. Therefore, we proposed a hypothesize that peripheral neutrophils probably elevated after SAH and the elevation of neutrophils count perhaps associated with unfavorable SAH clinical outcome. To identify a mechanistic pathway that may clarify the relationship between blood neutrophil count and neurological outcomes, we studied the role of NETs on microglia, which is the fundamental effector cells for inflammation response in the central nervous system [26, 27].

## METHODS

### Ethics statement

All experimental procedures were approved by the Ethics Committee of the Second Affiliated Hospital, Zhejiang University School of Medicine and conformed to the guidelines of the institutional guidelines. All of the patients in this study provided signed informed consent.

### Participant recruitment and biospecimen data obtainment

Peripheral blood levels of granulocytes (including neutrophils, eosinophils and basophils), monocytes and lymphocytes were obtained from retrospectively recruited patients with SAH due to a ruptured aneurysm in the anterior circulation, and traumatic SAH patients were excluded. Healthy subjects served as controls. The peripheral blood data were sampled within 24 hours and were sampled once in controls. The severity of SAH was determined by Hunt and Hess (HH) grade and the clinical outcomes were determined by the modified Rankin Scale (mRS). According to mRS, patients were divided into good outcome (mRS ≤ 2) and poor (mRS > 2) outcome groups.

### SAH model

An endovascular perforation SAH animal model was established in mice according to previous studies [28, 29]. Male C57BL/6 mice (8–10 weeks old) were purchased from SLAC Laboratory Animal Company, Shanghai, China. Mice were anesthetized using 2% isoflurane and ventilated with 1% isoflurane. Briefly, after exposing the carotid artery and its branches in the left side, a 5–0 sharpened monofilament nylon suture was advanced to the bifurcation of the anterior and middle cerebral artery. To produce SAH, Then, vascular perforation was implemented. For sham-operated mice, the same surgical procedure was implemented without vascular perforation.

### Study design and drug administration

Study design was showed in Supplementary Figure 1.

For neutrophil depletion, 50 μg of an anti-Ly6G antibody (Thermo Fisher) was intravenously injected into mice at 2 days before SAH [30].

DNAse I (50 μg in 250 μL of saline intraperitoneally and a second dose of 10 μg intravenously) or vehicle was injected 3 hours after SAH induction as previously described [31].

### Isolation of circulating neutrophils

As previously described, Histopaque (Sigma Aldrich) gradients were used to isolate neutrophil from mouse blood [32]. In short, the Histopaque 1117 (3 ml) was layered on Histopaque 1119 (3 ml) in a 15-ml tube, then the mouse blood (1 ml) was carefully placed on the top of the Histopaque mixture. After that, centrifugation at 400 g was performed by a swinging rotor for 30 minutes. The first ring containing monocytes was gently aspirated. The second ring containing neutrophils was moved to another 15-ml tube containing PBS. Then, centrifugation at 1500 g was performed for 10 minutes and the resulting pellet was suspended in 3 ml PBS and placed on Histopaque-1119 (3 ml). Then, centrifugation at 1500 g was performed again for 10 minutes at 4°C. Finally, the pellet including neutrophils was suspended for further use.

### Treatment of BV-2 cells with neutrophil from sham or vehicle-treated or DNase I-treated mice

**Figure 1:**
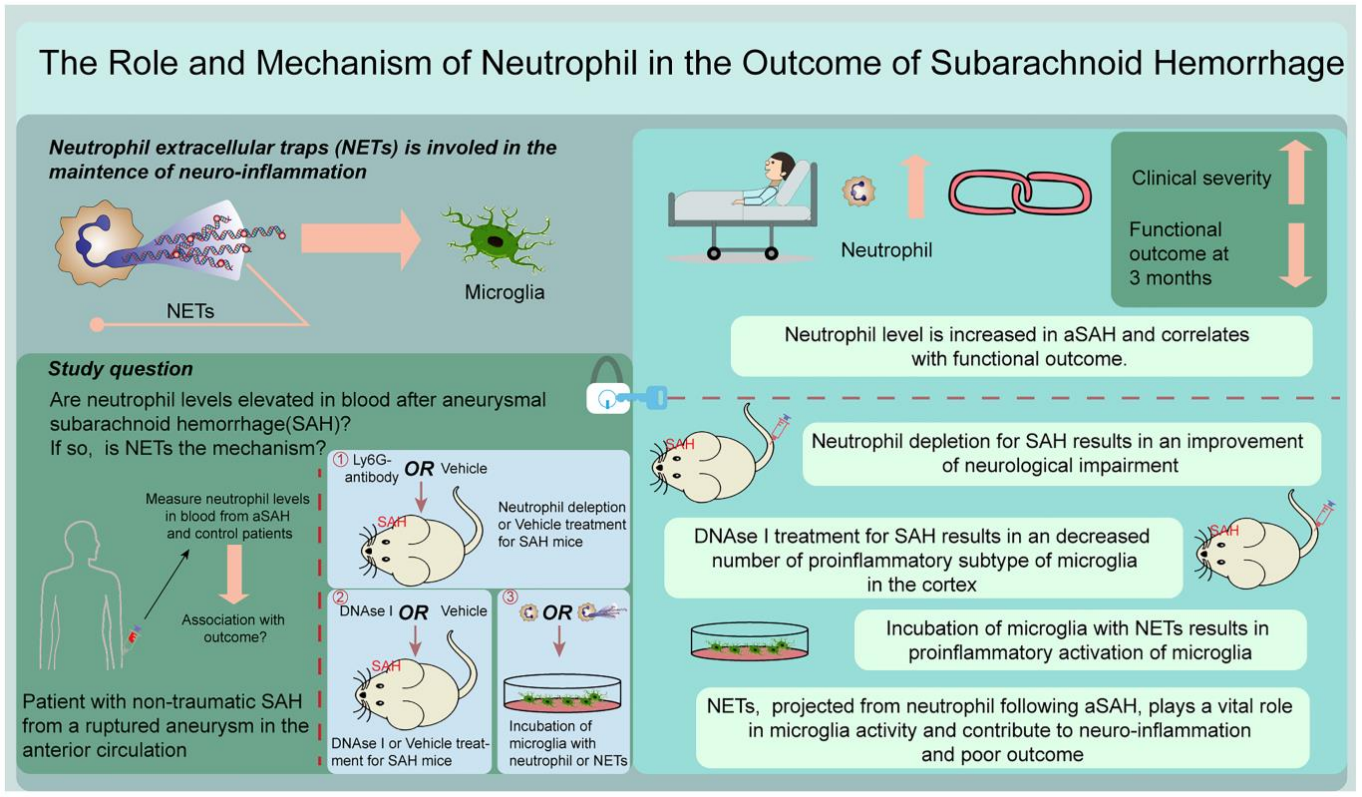
Graphical abstract.

BV-2 cells (4 × 10^5^/well) were treated in DMEM with 10% fetal bovine serum and antibiotics (100 U penicillin and 100 mg streptomycin/ml), and then were placed in a 37°C humidified incubator. Neutrophils from different groups were cultured with NMEM-conditioned media (NCM) for the indicated times (Figure 1). Then, NCM-treated neutrophils (5 × 10^5^/well) were cocultured with BV-2 cells using a Transwell co-culture device, and the pore size was 3 μm.

### SAH grade and neurological outcome scores

SAH grade was assessed at 24 h after SAH according to previous literature [33, 34]. The basal cistern of the brain was divided into six parts, with each part ranging from 0 to 3 points depending on the degree of blood coagulation. A total score was obtained by adding the scores from all six parts.

Neurological scores were evaluated at 24 h after SAH according to previous methods [33, 35]. Under a modification of the Garcia scoring system, the mice could receive possible scores ranged from 3 to 18. Another neurobehavioral test was also introduced [35]. The mice could receive possible scores ranged from 0 to 6. A higher modified Garcia score and a lower score in the other neurobehavioral test indicated a better neurological results. All of the tests were evaluated blindly.

### Brain edema

Brain edema was evaluated by measuring brain water content at 24 h after SAH [36]. Briefly, the left hemispheres were collected and weighed after immediate removal to get wet weight, and the left hemispheres were dried at 105°C for three days to get dry weight. Formula (brain water content = (wet weight − dry weight)/wet weight) was used to calculate brain water content.

### Flow cytometric analysis

Blood and BV-2 cells were prepared for fluorescence-activated cell sorting (FACS) after erythrolysis [30]. The antibodies followed were employed for blood: CD45-PerCp/Cy5.5 (103132, 1:400, Biolegend), CD11b-APC (101212, 1:400, Biolegend), and Ly6G-PE (127608, 1:400, Biolegend). The following antibody was used for BV-2 cells: CD16/32 (101307, 1:400, Biolegend). The percentage of neutrophils (the number of CD45+CD11b+Ly6G+ cells/the number of white blood cell (WBCs)) represents the changes in neutrophil levels in peripheral blood in mice after SAH. The median fluorescence intensity (MFI) of CD16/32 represents the changes in BV-2 cells cocultured with different media.

### Immunofluorescence staining

PMNs were isolated from peripheral blood and the slides were prepared with a cytospin, and BV-2 cells were immobilized by using a cell slide. Then, the slides were fixed with 4% paraformaldehyde for 15 min. The samples were blocked and incubated at 4°C overnight with the following antibodies: an anti-CitH3 (ab5103, Abcam), anti-MPO (ab90812, Abcam), and anti-CD16/32 (Cat# 553141, Biosciences).

Brains were isolated and fixed with 4% paraformaldehyde by transcardiac perfusion and then stored in the 4% paraformaldehyde overnight at 4°C. Then, brain samples were immersed in 30% sucrose until sinking to the bottom. And a cryostat was used to cut 8 μm-thick slices. The brain slides were blocked for 1 h and incubated at 4°C overnight with the following antibodies: anti-CitH3 (ab5103, Abcam), anti-NE (ab68672, Abcam), anti-Ly6g (ab25377, Abcam), anti-MPO antibody (ab90812, Abcam), anti-IBA-1 (ab48004, Abcam), anti-CD16/32 (Cat# 553141, Biosciences), and anti-IL-1β (ab9722, Abcam).

The corresponding fluorescence-conjugated secondary antibodies were then used at room temperature for 2 h, followed by staining with DAPI for 10 min. Finally, the sections were assessed with a fluorescence microscope. Fluoro-Jade C (FJC) staining was used to detect neuronal damage according to the manufacturer’s protocol (Roche Inc.). To detect the death of BV-2 cells, terminal deoxynucleotide transferase deoxyuridine triphosphate nick end labeling (TUNEL) staining was used according to the manufacturer’s protocol (Roche Inc.). All procedures were evaluated blindly.

### Western blot

Left cerebral cortex or BV-2 cells were collected for western blot. Briefly, an equal amount of protein from each sample was prepared and separated by an SDS-PAGE, and transferred to nitrocellulose membranes. After that, the membranes were blocked with nonfat dry milk buffer for 2 h, and then incubated at 4°C overnight with the following antibodies: anti-TNF-α (11948, CST) and anti-IL-1β (ab9722, Abcam). The membranes were incubated with secondary antibody for 1 h at room temperature. The results were detected by X-ray film and quantified by ImageJ software (National Institutes of Health).

### Statistical analysis

Statistical analysis was handled by GraphPad Prism and SPSS software. Continuous data are shown as the mean ± SD or median (interquartile range). For the data meeting normality, significant differences among groups were analyzed using Student’s *t*-test (2 groups) and one-way analysis of variance (ANOVA) (≥3 groups) followed by Tukey’s post hoc test. For the data failed to normality, significant differences among groups were analyzed using a nonparametric test (2 groups) or Kruskal-Wallis test (≥3 groups). Associations between continuous data were analyzed by Spearman correlation. Statistical significance was indicated at *P* < 0.05.

### Ethics approval

This study was approved by the Ethics Committee of the Second Affiliated Hospital, Zhejiang University School of Medicine, and was conducted in accordance with the principles of Good Clinical Practice and the Declaration of Helsinki. All of the patients in this study provided signed informed consent. All animal procedures were approved by the Animal Care and Use Committee of Zhejiang Medical University and were performed in accordance with institutional guidelines.

### Availability of data and materials

All raw data used in this manuscript are available on reasonable request.

### Consent to participate

All of the patients in this study provided signed informed consent.

## RESULTS

### Human participant characteristics

A total of 199 patients according to inclusion criteria and 20 healthy controls were included (Table 1).

**Table 1:** Patient characteristics and clinical features.

	**Controls (Healthy) (*n* = 20)**	**All SAH (*n* = 199)**	**3-month outcome**		
			**Good (≤2) (*n* = 141)**	**Poor (>2) (*n* = 43)**	**Null (*n* = 15)**
**Age, y**	60.3 (38–82)	56.4 (20–88)	55.6 (21–80)	60.1 (20–88)	52.7 (33–75)
**Sex, Male**	7 (35)	75 (38)	55 (39)	15 (35)	5 (33)
**Aneurysm location**					
**Acom** **IC-PC** **MCA** **Distal ICA** **Other ICA**		72 (36) 58 (29) 40 (20) 15 (8) 14 (7)	49 (35) 44 (31) 28 (20) 10 (7) 10 (7)	17 (40) 12 (28) 10 (23) 3 (7) 1 (2)	6 (40) 2 (13) 2 (13) 2 (13) 3 (20)
**HH grade**					
**1** **2** **3** **4** **5**		12 (6) 113 (57) 38 (19) 29 (15) 7 (3)	11 (8) 84 (60) 26 (18) 18 (13) 2 (1)	0 (0) 17 (40) 11 (25) 10 (23) 5 (12)	1 (7) 12 (80) 1 (7) 1 (7) 0 (0)
**Treatment**					
**Clip** **Coil**		123 (62) 76 (38)	85 (60) 56 (40)	28 (65) 15 (35)	10 (67) 5 (33)

Abbreviations: Acom: anterior communicating artery; HH: Hunt and Hess; IC-PC: internal carotid-posterior communicating artery; ICA: internal carotid artery; MCA: middle cerebral artery; SAH: subarachnoid hemorrhage. Values are mean (range) or *n* (%).

### Peripheral blood levels of neutrophils are elevated in SAH and negatively correlate with clinical outcome in patients

**Figure 2:**
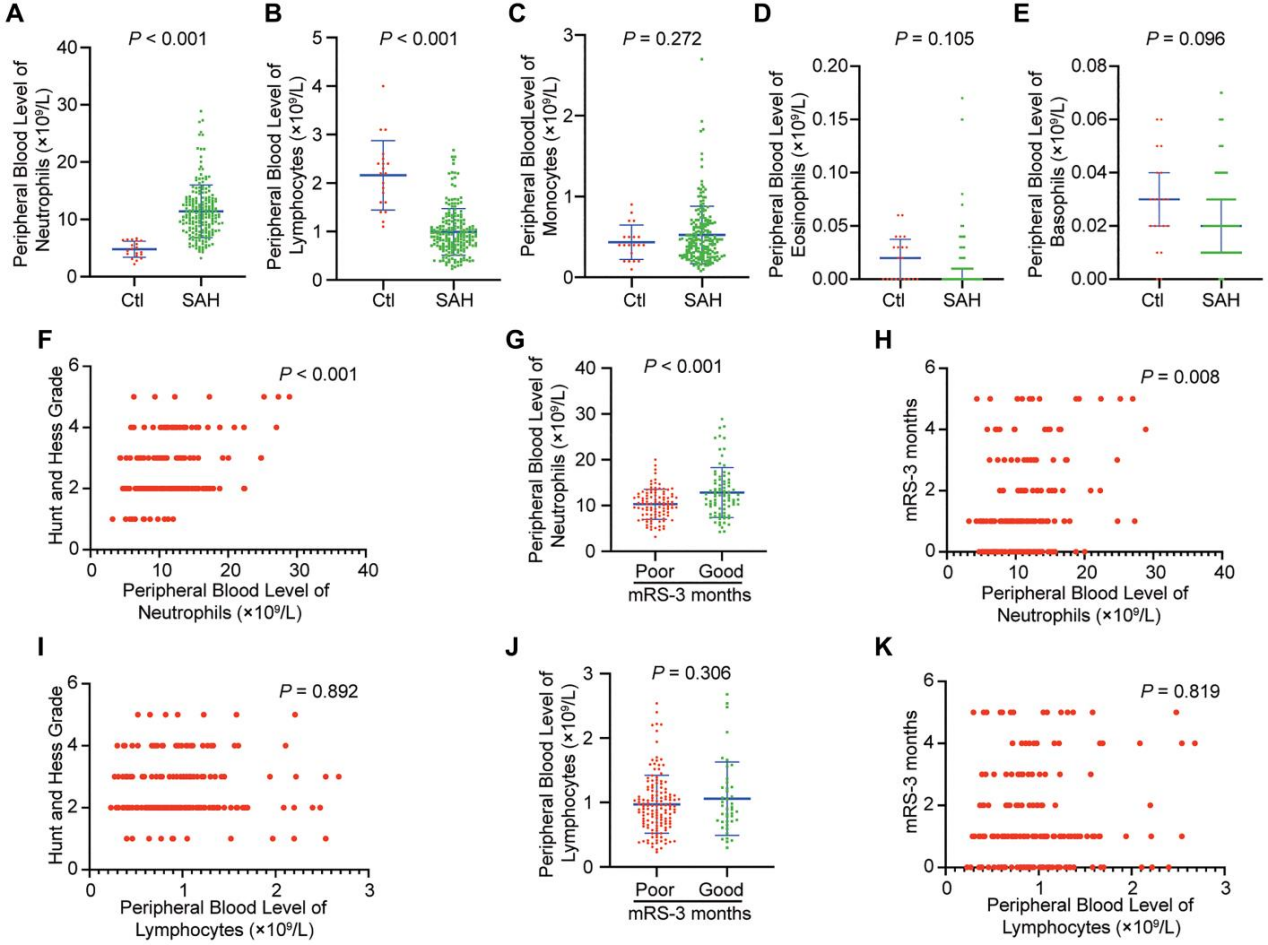
**Peripheral blood level of neutrophils is increased in SAH and correlates with functional outcome in patients.** (**A**) Peripheral blood level of neutrophils in patients with SAH within 24 hour compared to that of controls (*p* < 0.001, Student *t*-test). (**B**) Peripheral blood level of lymphocytes in patients with SAH within 24 hour compared to that of controls (*p* < 0.001, Student *t*-test). (**C**) Peripheral blood level of monocytes in patients with SAH within 24 hour compared to that of controls (*p* = 0.272, Student *t*-test). (**D**) Peripheral blood level of eosinophils in patients with SAH within 24 hour compared to that of controls (*p* = 0.105, Mann-Whitney test). (**E**) Peripheral blood level of basophils in patients with SAH within 24 hour compared to that of controls (*n* = 20) (*p* = 0.096, Mann-Whitney test). (**F**) Initial Hunt and Hess classification and peripheral blood level of neutrophils within 24 hours are positively correlated after SAH (*p* < 0.001, Spearman correlation). (**G**) Patients with SAH who developed poor outcome (modified Rankin Scale [mRS] > 2) at 3 months had higher peripheral blood level of neutrophils within 24 hours compared to those who had good outcome (mRS < 2) at 3 months (*p* < 0.001, Student *t*-test). (**H**) There is a positive correlation between mRS-3 months and peripheral blood level of neutrophils within 24 hours (*p* = 0.008, Spearman correlation). (**I**) Initial Hunt and Hess classification and peripheral blood level of lymphocytes within 24 hours are not positively correlated after SAH (*p* = 0.892, Spearman correlation). (**J**) Patients with SAH who developed poor outcome (modified Rankin Scale [mRS] > 2) at 3 months did not have significant change of peripheral blood level of lymphocytes within 24 hours compared to those who had good outcome (mRS < 2) at 3 months (*p* = 0.306, Student *t*-test). (**K**) There is not a positive correlation between mRS-3 months and peripheral blood level of lymphocytes within 24 hours (*p* = 0.819, Spearman correlation).

The serial peripheral blood levels of granulocytes (including neutrophils, eosinophils and basophils), monocytes and lymphocytes in included individuals were determined (Figure 2A–2E). The peripheral blood level of neutrophils was significantly higher in patients with SAH within 24 hours than in controls (Figure 2A; *p* < 0.001), and the peripheral blood level of lymphocytes was significantly lower (Figure 2B; *p* < 0.001). However, the levels of other granulocytes and monocytes show no significant changes. Higher neutrophil levels were associated with the severity of SAH (higher HH grade) (Figure 2F; *p* < 0.001). The higher peripheral blood level of neutrophils was detected in the poor outcome group at 3 months after SAH, when compared with good outcome group (Figure 2G; *p* < 0.001). An association was also found between neutrophil levels and clinical outcome at 3 months after SAH (Figure 2H; *p* = 0.008). However, lymphocyte levels indicated no significant association with SAH severity and outcome and no significant change in the different outcome groups (Figure 2I–2K; *p* > 0.05). These results suggested that neutrophils negatively correlated with functional outcome in SAH.

### Temporal patterns of neutrophils in peripheral blood and brain after SAH and neutrophil levels negatively correlate with neurological function in mice

**Figure 3:**
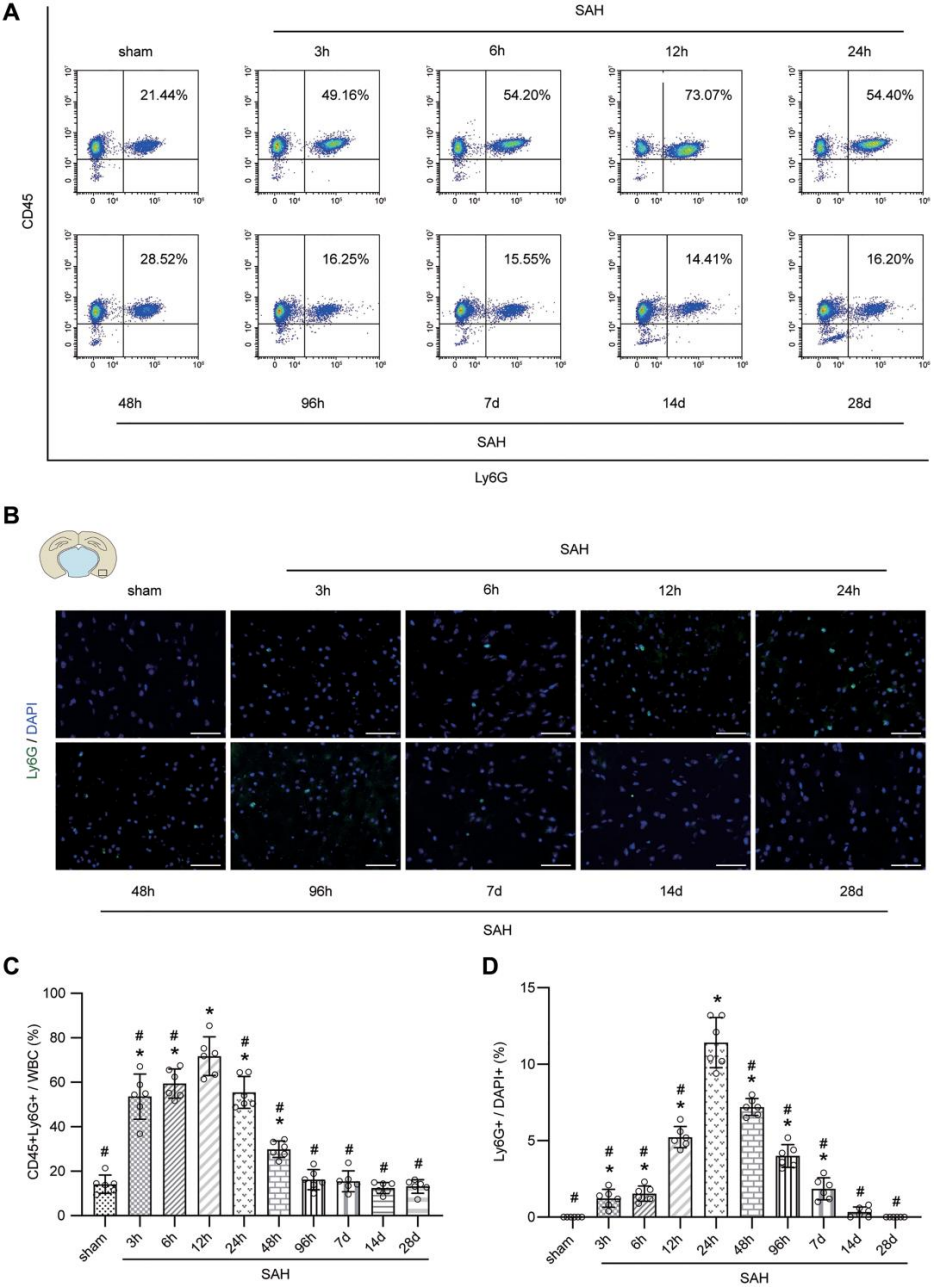
**Temporal patterns of neutrophils in peripheral blood and brain after SAH.** (**A**) Representative FACS of blood from sham or SAH mice in different temporal patterns and the percentage of PMNs. (**B**) Representative photographs of immunofluorescence staining showing neutrophils with Ly6G (green) in cortex from sham or SAH mice in different temporal patterns. (**C**) Quantitative analyses of the percentage of PMNs from blood in different temporal patterns. *n* = 6/group. ^*^p < 0.05 vs sham, ^#^*p* < 0.05 vs SAH-12 h. (**D**) Quantitative analyses of the percentage of neutrophils from cortex in different temporal patterns. *n* = 6/group. Scale bar = 50 μm. ^*^p < 0.05 vs sham, ^#^*p* < 0.05 vs SAH-24 h.

**Figure 4:**
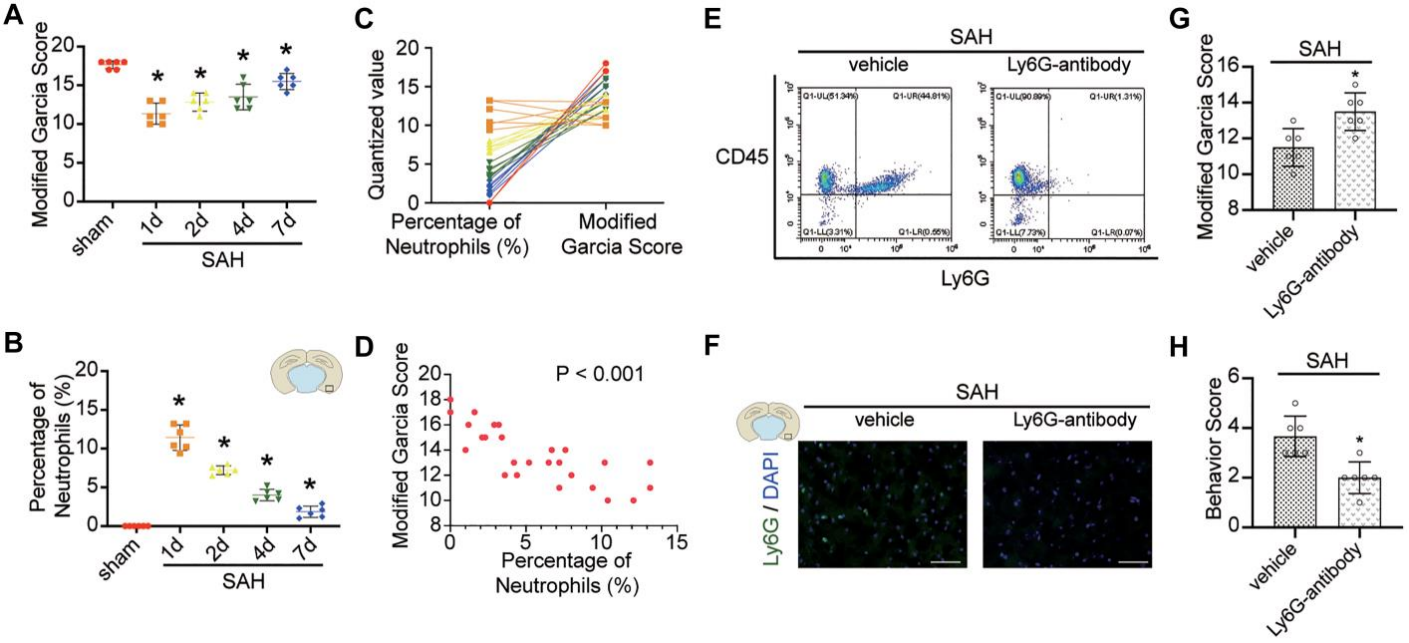
**Effect of neutrophils in brain after SAH.** (**A**) Modified Garcia Score from sham or SAH mice in different temporal patterns. (**B**) Quantitative analyses of the percentage of neutrophils from cortex in different temporal patterns. *n* = 6/group. ^*^p < 0.05 vs sham. (**C**) One to one correspondence diagram of Modified Garcia Score and the percentage of neutrophils from cortex. (**D**) There is a positive correlation between Modified Garcia Score and the percentage of neutrophils from cortex (*p* < 0.001). (**E**) Representative FACS of blood from SAH mice treated 2 days previously, without or with anti-Ly6G Ab (1A8) and the percentage of PMNs. (**F**) Representative photograph showed the neutrophils with Ly6G (green) in different groups. *n* = 5/group. Scale bar = 50 μm. (**G**) Quantification of Modified Garcia Score at 24 h after SAH. (**H**) Quantification of Behavior Score at 24 h after SAH. *n* = 6/group. ^*^P < 0.05 versus SAH + vehicle group.

The results of FACS and immunofluorescence staining demonstrated that the peripheral neutrophils and neutrophils in cerebral cortex were significantly increased after SAH. Furthermore, the level of neutrophils peaked at 12 h after SAH in peripheral blood and at 24 h in the cerebral cortex, after which the levels of neutrophils gradually declined (Figure 3A–3D; *p* < 0.05). We selected sham group mice and mice on days 1, 2, 4 and 7 after SAH for modified Garcia score evaluation, and the correlation analysis between the score results and the corresponding percentages of neutrophils in the cerebral cortex indicated that a significant negative correlation was existed between the two (Figure 4A–4D; *p* < 0.001).

### Benefit of neutrophils depletion after SAH

FACS and immunofluorescence staining showed the effectiveness of the anti-Ly6G antibody in the depletion of neutrophils (Figure 4E, 4F). Depletion of neutrophils reduced neurological injury compared with saline treatment in mice after SAH (Figure 4G, 4H; *p* < 0.05).

### The formation of NETs and proinflammatory subtype transition of microglia after SAH

**Figure 5:**
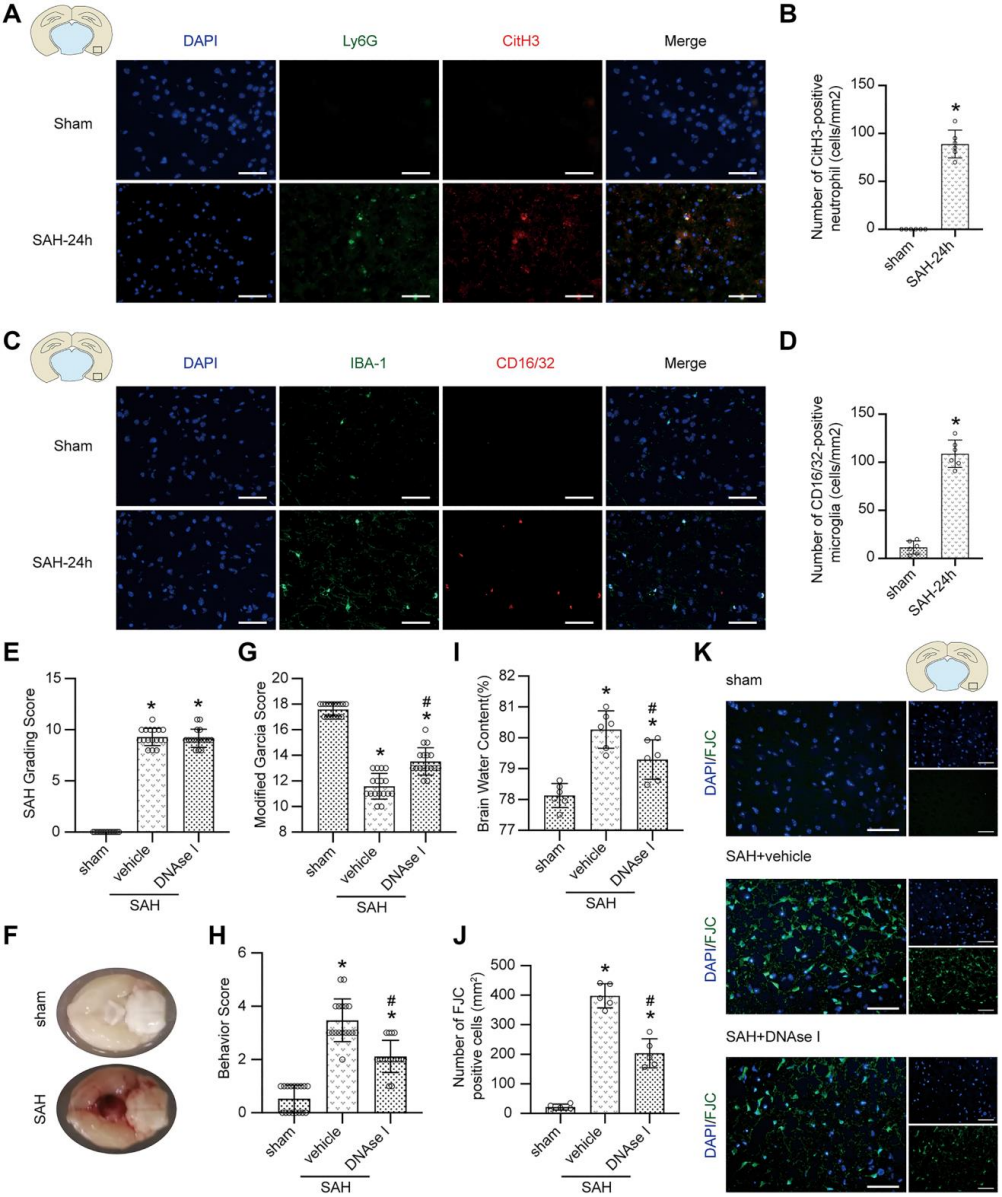
**The effect of the formation of NETs after SAH.** (**A**) Representative photographs of immunofluorescence staining showing NETs (marked with CitH3 (red)) in neutrophil (marked with Ly6G (green)) in sham group and SAH 24 h group. (**B**) Quantitative analysis of CitH3 positive neutrophils. *n* = 5/group (**C**) Representative photographs of immunofluorescence staining showing pro-inflammatory subtype (marked with CD16/32 (red)) microglia (marked with IBA-1 (green)) in sham group and SAH 24 h group. (**D**) Quantitative analysis of CD16/32 positive microglia. *n* = 5/group. Scale bar = 50 μm. ^*^P < 0.05 versus Sham. (**E**) The quantification of SAH grade. *n* = 17/group. (**F**) Typical brains without or with SAH. (**G**) Quantification of Modified Garcia Score at 24 h after SAH. *n* = 17/group. (**H**) Quantification of Behavior Score at 24 h after SAH. *n* = 17/group. (**I**) Quantification of brain water content. *n* = 6/group. (**J**) Quantitative analysis of FJC staining. *n* = 5/group. (**K**) Representative photograph showed the FJC positive cell (green) in different groups. Scale bar = 50 μm. ^*^P < 0.05 versus Sham, ^#^*P* < 0.05 versus SAH + vehicle group.

The immunofluorescence staining results demonstrated that the levels of CitH3 (a marker of NETs) and CD16/32 (a marker of proinflammatory subtype microglia) were significantly increased 24 h postmodeling in the brain (Figure 5A–5D; *p* < 0.05).

### Inhibiting the formation of NETs prevents neurological impairment after SAH

No significant difference was found in SAH grade among the modeling groups (Figure 5E; *p* > 0.05), and typical brains without or with SAH are shown (Figure 5F). After DNAse I treatment, a neuroprotective effect, including attenuated neurological impairment and alleviated brain edema, was shown (Figure 5G–5I; *p* > 0.05). Moreover, FJC staining also showed that the pharmacological elimination of NETs with DNAse I decreased the number of FJC-positive cells (Figure 5J, 5K; *p* < 0.05).

### Inhibiting the formation of NETs prevents proinflammatory subtype transition of microglia

**Figure 6:**
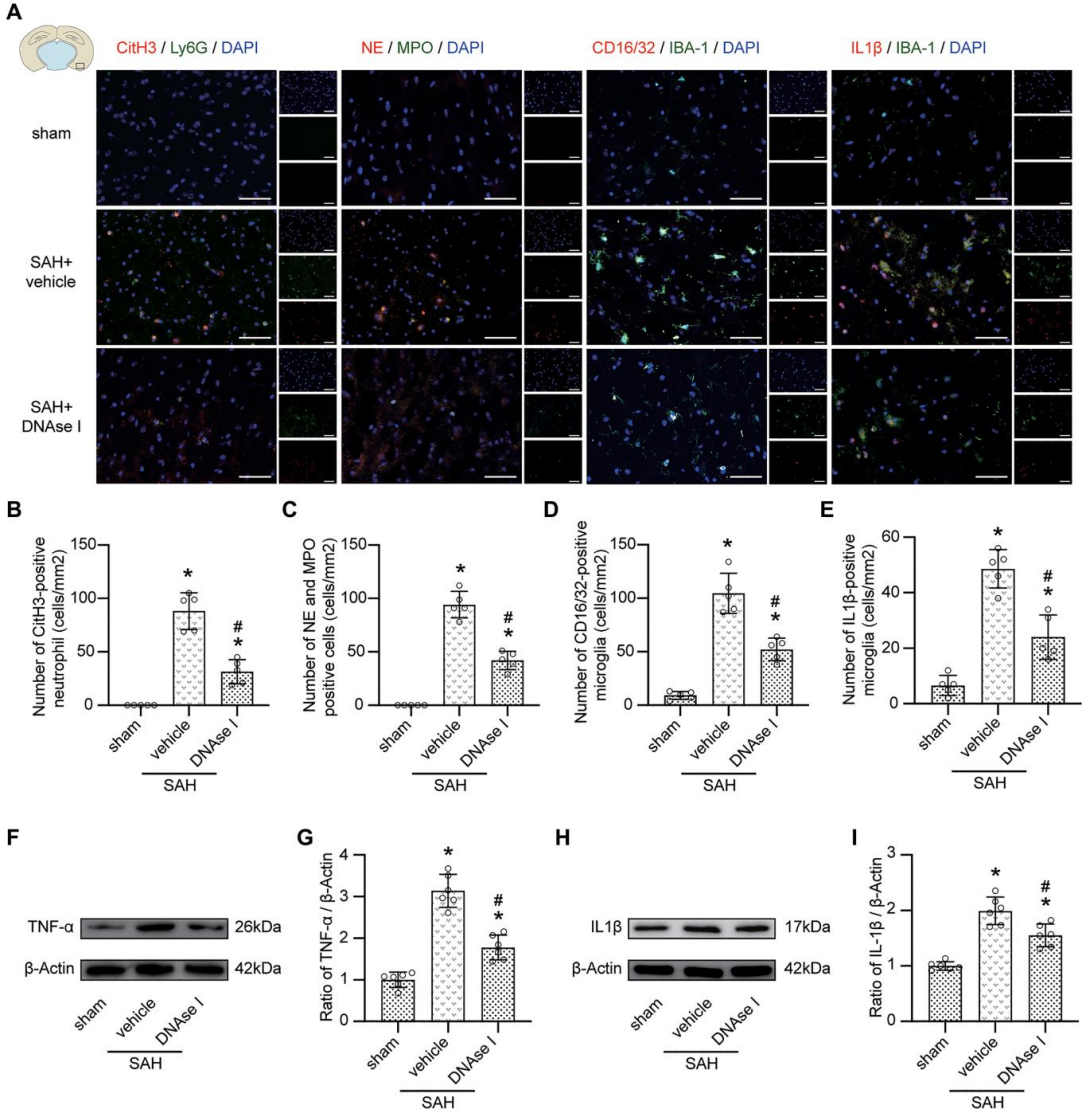
**Inhibiting the formation of NETs prevents pro-inflammatory subtype transition of microglia.** (**A**) Representative photograph showed the co-localization of CitH3 positive cell (red) with Ly6G (green), NE positive cell (red) with MPO (green), CD16/32 positive cell (red) with IBA-1 (green), IL1β positive cell (red) with IBA-1 (green), in different groups. (**B**) Quantitative analysis of CitH3 positive neutrophil in different groups. *n* = 5/group. (**C**) Quantitative analysis of NE and MPO positive cells in different groups. *n* = 5/group. (**D**) Quantitative analysis of CD16/32 positive microglia in different groups. *n* = 5/group. (**E**) Quantitative analysis of IL1β positive microglia in different groups. *n* = 5/group. (**F**) Representative western blotting images of TNF-α expression in cortex in different groups. (**G**) Quantitative analysis of TNF-α expression in cortex in different groups. *n* = 6/group. (**H**) Representative western blotting images of IL1β expression in cortex in different groups. (**I**) Quantitative analysis of IL1β expression in cortex in different groups. *n* = 6/group. Scale bar = 50 μm. ^*^P < 0.05 versus Sham, ^#^*P* < 0.05 versus SAH + vehicle group.

Immunofluorescence staining data indicated that compared with the SAH + vehicle group, the level of NETs (marked with CitH3, NE, and MPO) in the SAH + DNAse I group was significantly decreased (Figure 6A–6C; *p* < 0.05), and the level of proinflammatory subtype transition of microglia (marked with CD16/32, IL1β) was significantly decreased (Figure 6A, 6D, 6E; *p* < 0.05). Moreover, the upregulated level of TNF-α and IL-1β was also attenuated under DNAse I treatment (Figure 6F–6I; *p* < 0.05).

### The promoting role of NETs on proinflammatory subtype transition of BV-2 cells

**Figure 7:**
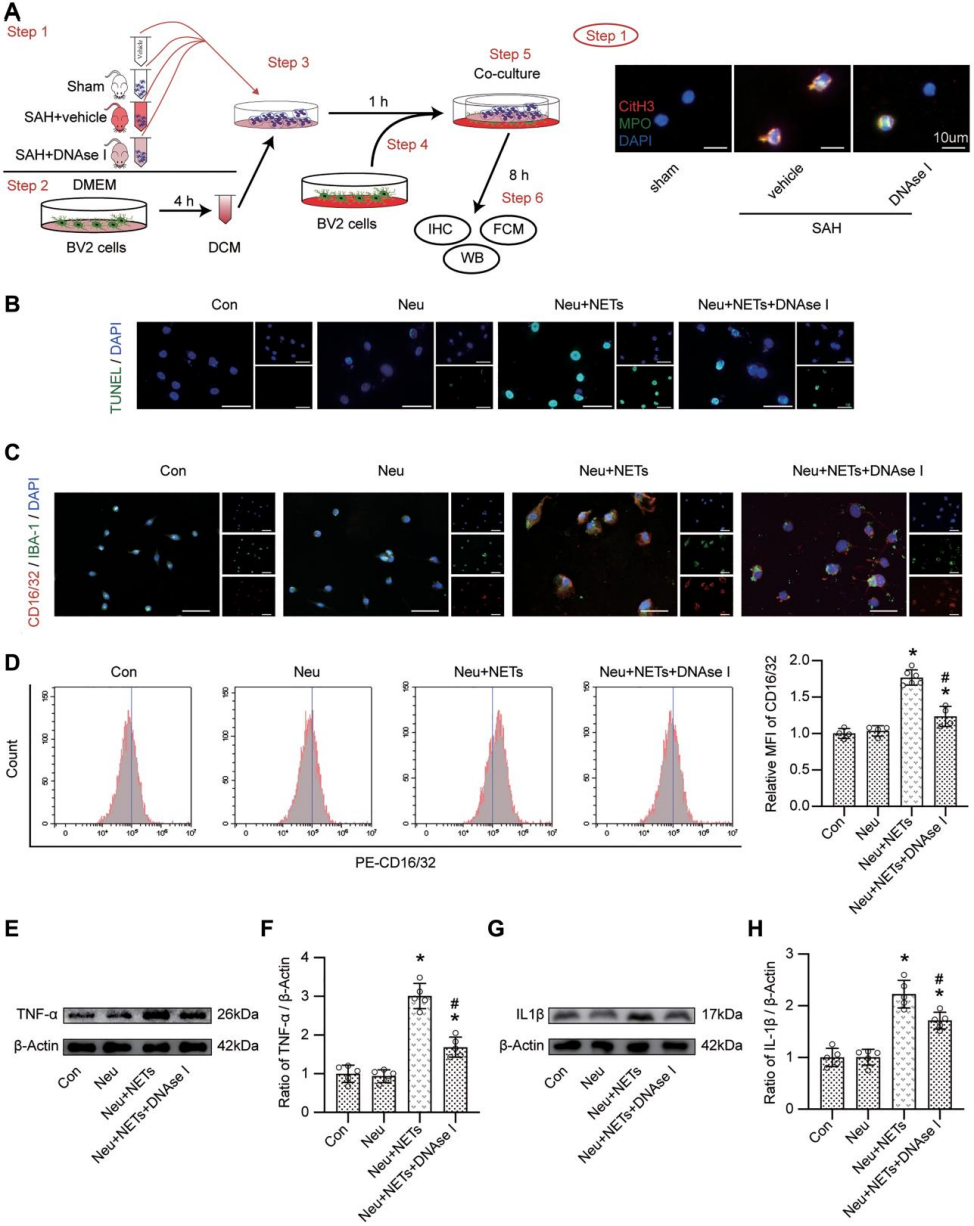
**The role of NETs on pro-inflammatory subtype transition of BV-2 cells.** (**A**) Study design *in vitro* and representative pictures of neutrophil from different mice. Scale bar = 10 μm. (**B**) Representative pictures of TUNEL positive cells in different groups. Scale bar = 50 μm. (**C**) Representative pictures of CD16/32 positive BV-2 cells in different groups. Scale bar = 50 μm. (**D**) Representative pictures of CD16/32 positive cells from FACS and Quantitative analysis of MFI of CD16/32. *n* = 4/group in Con, Neu and Neu+NETs+DNAse I groups, *n* = 6/group in Neu+NETs group. (**E**) Representative western blotting images of TNF-α expression in different groups. (**F**) Quantitative analysis of TNF-α expression in different groups. *n* = 5/group. (**G**) Representative western blotting images of IL1β expression in different groups. (**H**) Quantitative analysis of IL1β expression in different groups. *n* = 5/group. ^*^P < 0.05 versus Neu, ^#^*P* < 0.05 versus Neu+NETs group.

BV-2 cells were cocultured with vehicle with or without neutrophils from sham, vehicle-treated or DNAse I-treated mice to evaluate the effect of NETs on microglia. We then confirmed the changes in neutrophils from different groups (Figure 7A). After coculturing, TUNEL staining indicated an increase in the number of TUNEL-positive cells in the Neu+NETs group compared with the Con or Neu group. However, in the Neu+NETs+DNAse I group, the number of TUNEL-positive cells decreased (Figure 7B). Data from immunofluorescence staining indicated a similar change in CD16/32 (Figure 7C). Furthermore, FACS presented a significantly increased MFI of CD16/32 in the Neu+NETs group compared with the Con or Neu group. However, in the Neu+NETs+DNAse I group, the MFI decreased (Figure 7D, *p* < 0.05). In addition, the upregulation of TNF-α and IL1β was remarkably attenuated when the cells were cocultured with neutrophils from DNAse I-treated mice (Figure 7E–7H, *p* < 0.05).

## DISCUSSION

In this study, we identified a biological process in which the levels of peripheral neutrophils are elevated in SAH patients and are associated with unfavorable clinical outcome after SAH. This phenomenon led us to hypothesize that during SAH, neutrophils increase reactively in peripheral blood and infiltrate brain tissue with possible deleterious outcomes. After neutrophil depletion, neurological damage after SAH was relieved. Then, to identify a possible mechanism, we performed *in vitro* and *in vivo* experiments. Exposure to NETs activated the proinflammatory subtype transition of microglia and increased their inflammatory cytokine levels. Inhibiting the formation of NETs prevented these phenomena. In brief, our experimental findings may be consistent with clinical observations. NETs released from neutrophils after SAH contribute to the pathophysiology of neuroinflammation in SAH during the early phase.

We hypothesized that peripheral immune cells would have clinical significance in SAH. Nevertheless, our study only detected a difference in neutrophils and lymphocytes. Furthermore, correlation analysis showed that only neutrophils had a significant correlation with disease severity and prognosis. However, the numerous other clinical studies have indicated that the neutrophil-lymphocyte ratio may be a more sensitive and specific indicator for the prognosis of SAH [37, 38]. From these clinical studies, we speculated that both neutrophils and lymphocytes may involve in the pathogenesis of SAH. The clinical data used for analysis in our study were all untransformed data and were limited by the sample size. This may be the reason why we only found that neutrophils were positively related to disease severity and poor prognosis.

The negative role of neutrophils in SAH has been mentioned in many published articles, including studies on memory regulation, and cortical blood flow regulation [39, 40]. However, it is the first report of NETs, released from neutrophils contributing to the proinflammatory subtype transition of microglia after SAH. The NET-microglia interaction may be a constituent factor to persistent neuroinflammation. The current study support a new role of neutrophils that NETs may induce alteration of microglia into a proinflammatory subtype, thereby promoting neuroinflammation. However, NETs have been reported to have a second role in targeting senescent vasculature for tissue remodeling [9]. The involvement of a proinflammatory subtype transition is consistent with previously reported shifts in the macrophage response to NETs [41, 42]. Studies have shown that NETs have adverse effects on neurons and the blood-brain barrier, and then cause adverse consequences in central nervous system diseases [32, 43]. However, the relationship between NETs and microglia has not been well described. Our data suggest that NETs released from neutrophils may not only play a direct proinflammatory role but may also themselves shift into a proinflammatory state.

DNAse I reduces the NET structure by degrading chromatin fibers [44, 45]. Although the mechanisms include reducing toll-like receptor activation by circulating cell-free DNA, it is feasible that DNase I reduces brain injury by degrading NETs. In the present study, we resolved neurological damage after SAH by degrading NETs. Other than the direct effects of the reduction in NETs, there are existence of indirect benefits. One example is that neuroinflammation in the brain also promotes neutrophil recruitment, further aggravating inflammation [21, 46–49]. This may provide a logical connection between the inhibition of brain inflammation in SAH mice and the decreased neutrophil level in SAH mice after DNAse I administration.

There are some possible directions to explore in the future. First, how the other actions of neutrophils, including phagocytosis and degranulation, work in SAH needs to be explored. Second, whether microglial activation is counterproductive to neutrophils needs to be studied. Third, how neutrophils increase in peripheral blood after SAH should be investigated. Fourth, whether lymphocytes play a definite role in the brain after SAH needs to be explored. If these questions are answered, the research on neutrophils and SAH would be further improved. Of course, there are several limitations to our study. First, the number of cases in our series was limited. Second, the mechanisms by which neutrophil induce NETs require further investigation. Third, the clinical feasibility and translational potential of our findings demand more studies.

## CONCLUSIONS

NETs, released from neutrophils after SAH, can induce alteration of microglia into a proinflammatory subtype and contribute to neuroinflammation and poor outcome in subarachnoid hemorrhage.

## Supplementary Materials

Supplementary Figure 1
