# Supplementary material for: Neutrophil extracellular traps, released from neutrophil, promote microglia inflammation and contribute to poor outcome in subarachnoid hemorrhage
Source: Aging (Albany NY). 2021 May 8;13(9):13108–23. doi: 10.18632/aging.202993 (PMC8148446; doi:10.18632/aging.202993)
Supplement: Supplementary Figure 1 [file aging-13-202993-s001.pdf]

SUPPLEMENTARY FIGURE

A

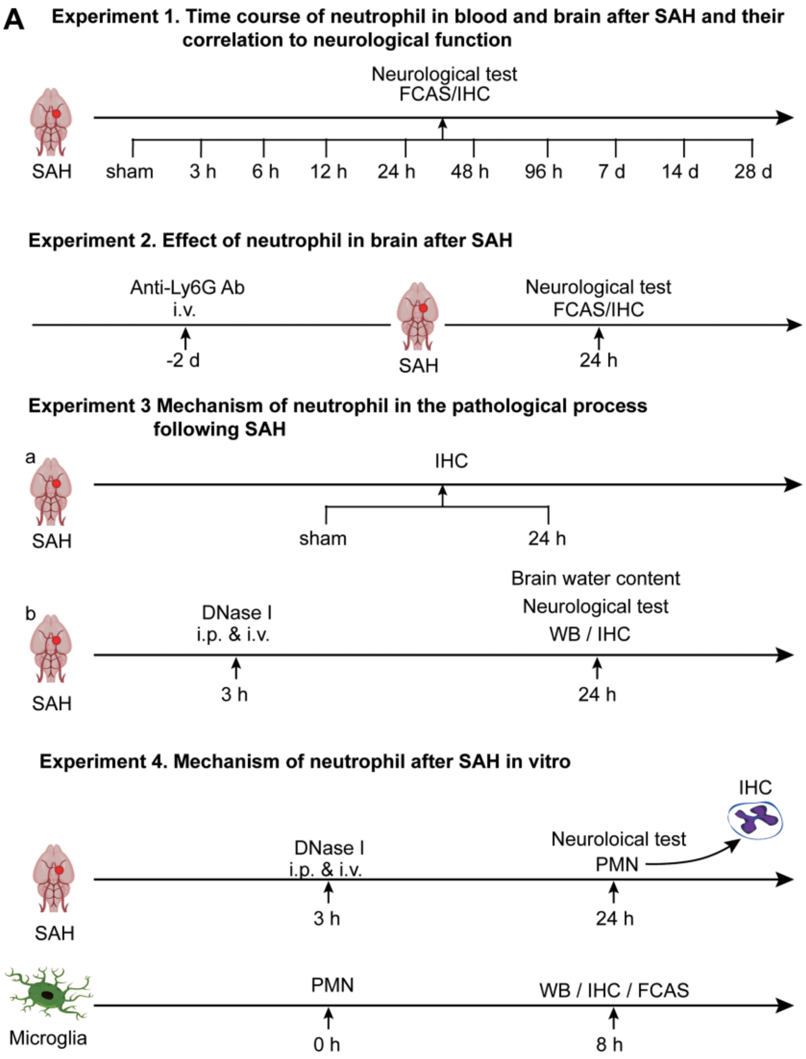

B

Experiments in vivo

Groups	Mortality rate
**Experiment 1, 3a**	
Sham	0.0% (0/6)
SAH (3h, 6h, 12h, 24h, 48h, 96h,7d, 14d, 28d)	22.9% (16/70)
**Experiment 2**	
SAH+vehicle	14.3% (1/7)
SAH+Ly6G-antibody	14.3% (1/7)
**Experiment 3b, 4**	
Sham	0.0% (0/29)
SAH+vehicle	22.5% (9/40)
SAH+DNAse I	19.4% (7/36)
**Total**	
Sham	0.0% (0/35)
SAH	21.3% (34/160)

C

Experiments in vitro

Groups
**Experiment 4**
Con
Neu
Neu + NETs
Neu + NETs + DNAse I

Supplementary Figure 1. Animal and cell experiment design, grouping, and animal mortality.
